# The diversity rank-score function for combining human visual perception systems

**DOI:** 10.1007/s40708-016-0037-3

**Published:** 2016-02-15

**Authors:** Christina Schweikert, Darius Mulia, Kilby Sanchez, D. Frank Hsu

**Affiliations:** 1 Division of Computer Science, Mathematics and Science, St. John’s University, Queens, NY USA; 2 Laboratory of Informatics and Data Mining, Department of Computer and Information Science, Fordham University, New York, NY USA

**Keywords:** Cognitive diversity, Combinatorial fusion analysis, Diversity rank-score function, Multiple scoring systems, Rank-score characteristic (RSC) function

## Abstract

There are many situations in which a joint decision, based on the observations or decisions of multiple individuals, is desired. The challenge is determining when a combined decision is better than each of the individual systems, along with choosing the best way to perform the combination. It has been shown that the diversity between systems plays a role in the performance of their fusion. This study involved several pairs of people, each viewing an event and reporting an observation, along with their confidence level. Each observer is treated as a visual perception system, and hence an associated scoring system is created based on the observer’s confidence. A diversity rank-score function on a set of observation pairs is calculated using the notion of cognitive diversity between two scoring systems in the combinatorial fusion analysis framework. The resulting diversity rank-score function graph provides a powerful visualization tool for the diversity variation among a set of system pairs, helping to identify which system pairs are most likely to show improved performance with combination.

## Introduction

The concept of multiple scoring systems has been applied to a variety of domains [1, 2]. In situations where multiple scoring systems are constructed, we are interested in conducting a meta-analysis to gain an understanding of the relationship between the systems, specifically the diversity between them. It has been shown that the combination of two scoring systems can outperform individual systems when there is some diversity between the systems, and they are of relatively good performance [1, 3]. To this end, quantitative measures of diversity can be used to generate diversity scores for pairs of systems, which can then be analyzed within the combinatorial fusion analysis (CFA) framework [1].

Human beings are constantly and naturally performing *fusion* of information within and among the senses. There is extensive research in this area on the neurological level pertaining to how fusion in the sensory system works [4–6], how visual information is combined with information from other senses [7–11], and how visual systems are combined [12, 9, 13]. In this study, however, we are focused on the inter-human level of information and fusion of the information at the decision level.

There are many situations in which two people’s observations are considered for a decision, such as referees in a football or tennis match, physicians examining a patient, co-pilots navigating a plane, and so on. For example, when two physicians are examining a new patient, each may observe different symptoms that can indicate different diseases; interactive consultation may lead to a final diagnosis. When two people are interactively making a decision based on visual input, research by Bahrami et al [12], Ernst and Banks [7], and Kepecs et al [13] suggests that these decisions are improved when two people are interactively making the decision, rather than an individual. The question then becomes, if we have two people making visual observations of an event, how do we integrate these observations or decisions? Do we choose one of the observer’s results, or create a combination of the two? Koriat [14] emphasizes the importance of confidence, and that it may be a good option to take the decision of the more confident person. The approach taken in our study is to combine the observations or decisions made by two people in an attempt to outperform the individual decisions. The visual observations tested in this project involve pairs of volunteers that are asked to give the location of a small object they observe being tossed in a field.

In order to perform the desired combination, by score or by rank, a scoring system must first be constructed for each participant in a trial. Each participant’s observation, or perception system, is represented as a scoring system, which is made up of a score function and a rank function. Given this multiple scoring system scenario, we then analyze the cognitive diversity between the scoring systems of a trial. A quantitative diversity measure, the distance between two rank-score functions, is used to represent the cognitive diversity between two scoring systems [1, 2]. Examining the relative diversities between the system pairs, together with the performance of their combinations, can give us insight into how diversity variation may play a role in the performance of system combinations. The diversities between systems are analyzed using the diversity rank-score functions, which are then visualized in diversity rank-score graphs. This visualization of diversity variation is beneficial in situations where there are a large number of scoring system pairs (hundreds or thousands). Interactive data visualization [15–17] is a dynamic field in which data are visualized with the intent to facilitate an end user in a particular task. The diversity rank-score function graph is such a tool that has potential to be integrated into various data analytics and software systems.

Information fusion can be applied to many situations where there are multiple scoring systems, or multiple classifiers. For example, the CFA framework [18, 1, 2] has been applied to information retrieval [19], text categorization [20], target tracking [21], sensor feature selection and combination [22], and image skeleton pruning [23]. Combinatorial fusion has also been used for enhancing the analysis of various biomedical datasets including virtual screening for molecular compounds [3], protein structure prediction [24], and ChIP-seq peak detection [25]. When combining multiple models (performing information fusion), it would be useful to know in advance whether the fusion will outperform the best model. Ng and Kantor [26] identify system features that can help predict whether fusion will be beneficial. Combination of multiple classifiers has also been shown to improve results in the area of pattern recognition. [27, 28]

The content of this paper is organized as follows: Sect. 2 describes the concept of multiple visual perception systems, along with the corresponding multiple scoring systems, which are considered a generalization of multiple classifier systems. The CFA framework, which establishes each visual perception system as a scoring system and combines two such systems, is also described. The diversity rank-score function can be used as a guiding light to combine pairs of visual perception systems based on the diversity variation across a set of trials. In Sect. 3, we describe the visual perception dataset, present the results of scoring system combinations, and examine the role of the diversity rank-score function graph in the context of diversity variation and visualization. Concluding remarks and discussion are included in Sect. 4.

## Multiple visual perception systems

### From multiple classifier systems to multiple scoring systems

In many domains, such as biomedical informatics, finance, security, information retrieval, among others, classification models are created in order to generate class predictions for new data. Binary classifiers attempt to categorize items into one of two classes (or labels). For example, determining whether a webpage is relevant to a search term or not, or whether a patient tests positive or negative for a disease. Some binary classification problems are asymmetric, meaning one class occurs much less frequently than the other. Multiclass classifiers involve more than two classes.

The output of a classification system includes a class prediction, along with an associated probability. Treating these probabilities as scores, and sorting the results by score to generate rankings, enables us to consider classification systems as a scoring system that have a score function and a rank function.

In an effort to improve classification accuracy, it is often desired to incorporate the results from multiple classifiers that are varied in terms of their approach or algorithm. The element of variety, or diversity, is essential since different classifiers may contribute various perspectives, results, or predictions, on the data. Generally, the results from multiple classifier systems are combined using ensemble methods such as majority voting (bagging) or weighted voting (boosting). Table 1a, b contains a snapshot from a classification example in which the class label of a sample document is predicted in each of the following two cases: (a) 3 class labels, and (b) 6 class labels (Table 1a, b). The document is analyzed by 4 different classifiers, each of which output the probability that the document belongs to class A, B, or C, in the case of 3 class labels. In Table 1b, each document belongs to one of the 6 class labels: A, B, C, D, E, or F. For each classifier, the class label with the highest probability is considered the predicated class label and is assigned rank 1. Likewise, the next highest probability is assigned rank 2, and so on. The ensemble approach of majority voting is used to combine the results of the individual classifiers. For each class label, we count the number of times that class is ranked 1 (has the highest probability) by a classifier. Then, the class label with the highest number of votes is considered the predicted class for the document.

If we consider the classifiers as scoring systems (see Table 2), we can apply score and rank combinations as an alternative *ensemble* approach. Here, the probabilities are treated as scores, which are then ranked. Score combination (SC), in this example, is the average of the scores for a class label across the 4 classifiers. The class label with the highest average score is chosen as the result. The rank combination (RC) is computed as the average rank for a class label for all classifiers. The class label with the lowest average rank is then selected. Weighted averages can be used if the past performance of the classifiers is known. In this example, we can see that combining by score or rank may produce different results. Table 1b is a classification problem that involves more possible class labels. In this example, we see that classifiers can be viewed as *scoring systems*, where the scores are the class label probabilities. The concept of multiple classifier systems with multiple class labels (the case in Table 1b) is then generalized to multiple scoring systems with multiple choices (items or options) (as is the case in Table 2).

When constructing an ensemble, it is desired to have diversity among the component classifiers or scoring systems. Several techniques for measuring diversity have been proposed for regression and classification [29, 30]. It is more challenging to measure diversity between classifiers if we just consider the output class labels, without their associated probabilities [29].

Viewing classification systems as scoring systems enables us to apply the concept of diversity that has been defined for multiple scoring systems [1, 2, 26, 3].

**Table 1:** Combination of multiple classifier systems with (a) 3 class labels and (b) 6 class labels using majority voting (*C*_*MAJ*_), score combination (*C*_*SC*_), and rank combination (*C*_*RC*_)

a							
Classifier	*C*_1_	*C*_2_	*C*_3_	*C*_4_	*C*_*MAJ*_	*C*_*SC*_	*C*_*RC*_
Class label							
A(score, rank)	(0.74, 1)	(0.05, 3)	(0.55, 1)	(0.31, 3)	2	0.41	2
B(score, rank)	(0.14, 2)	(0.48, 1)	(0.25, 2)	(0.33, 2)	1	0.30	1.75
C(score, rank)	(0.12, 3)	(0.47, 2)	(0.20, 3)	(0.36, 1)	1	0.29	2.25
Class label	A	B	A	C	A	A	B

b											
Classifier	*C*_1_		*C*_2_		*C*_3_		*C*_4_		*C*_*MAJ*_	*C*_*SC*_	*C*_*RC*_
Class label	s	r	s	r	s	r	s	r			
A	0.32	1	0.05	3	0.03	6	0.30	1	2	0.18	2.75
B	0.11	4	0.04	4	0.25	2	0.11	4	0	0.13	3.5
C	0.01	6	0.03	5	0.45	1	0.08	5	1	0.14	4.25
D	0.27	2	0.81	1	0.04	5	0.22	3	1	0.34	2.75
E	0.05	5	0.01	6	0.11	4	0.06	6	0	0.06	5.25
F	0.24	3	0.06	2	0.12	3	0.23	2	0	0.16	2.5
Class label	A		D		C		A		A	D	F

**Table 2:** Combining multiple scoring systems (with 3 scoring systems) to rank a set of items (with 8 items)

	*J*_1_		*J*_2_		*J*_3_		s(SC)	r(SC)	s(RC)	r(RC)
	s	r	s	r	s	r				
*d*_1_	8.5	4	7	5	9.7	4	25.2	4	13	4.5
*d*_2_	7.6	7	8.4	3	9.6	6	25.6	3	16	7
*d*_3_	8.3	5	5.6	7	9.75	3	23.65	7	15	6
*d*_4_	6.4	8	7.4	8	9.81	2	21.61	8	18	8
*d*_5_	9.4	3	7.8	4	9.68	5	26.88	2	12	3
*d*_6_	9.5	2	8.5	2	9.2	7	27.2	1	11	2
*d*_7_	7.9	6	6.3	6	10	1	24.2	6	13	4.5
*d*_8_	10	1	10	1	5.1	8	25.1	5	10	1

### The combinatorial fusion framework

#### Establishing each visual perception system as a scoring system

In situations where we have a set of documents (webpages, genes, customers, etc.) that are assigned scores or probabilities by an algorithm or classifier, creating a scoring system is straightforward. However, in cases where we do not have a set of scores to work with, a score function needs to be generated based on the value(s) given. In this experiment, when an observer is deciding on the proposed landing point of the object based on the visual input, he/she is selecting from several locations within a range. Intervals within this visual range will be considered as the items (or options) that will be scored and ranked. Since there are two subjects within each trial, the corresponding score functions must score the same set of intervals. To this end, a common visual space is created, as described in previous work [18]. First, the mean of the decisions (points) for the two observers P and Q is computed in three different versions, varying the weight given to the confidence radius *σ*. $M_0, M_1$, and * M*_2_, are computed as

1 $$M_i = \frac{\frac{P}{\sigma _1^i}+\frac{Q}{\sigma _2^i}}{\frac{1}{\sigma _1^i}+\frac{1}{\sigma _2^i}}$$

**Fig. 1:**
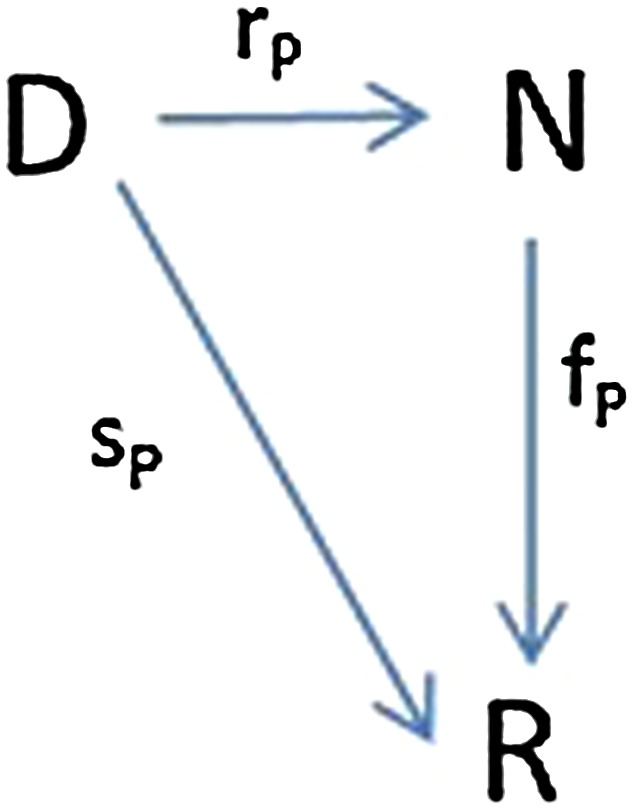
Scoring system P with: (a) score function $s_P$, (b) rank function $r_P$, and (c) rank-score characteristic (RSC) function $f_P$

The scoring system analysis is performed for each version of * M*_*i*_. Specifically, the * M*_*i*_ values are used as a foundation point from which to create a common visual space. The * M*_*i*_ points are always located between the P and Q original points. The visual space is also extended on both sides of P and Q. The common visual space is divided into 63 intervals. The interval scores are computed using a normal distribution around * M*_*i*_, using the confidence radius (0.5r) for the standard deviation. The performance of each * M*_*i*_ is measured as the distance from * M*_*i*_ to the actual location of the object [31]. The scores, created for the intervals for P and Q, give us the score functions $s_P$ and $s_Q$. Given a set of intervals ${d_1,d_2,\ldots ,d_n}$, the scoring system P consists of a score function $s_P$, rank function $r_P$, and rank-score characteristic (RSC) function $f_P$ (see Fig. 1). The rank function for the scoring systems P and Q are obtained by sorting $s_P$ and $s_Q$ and assigning ranks to create the rank functions $r_P$ and $r_Q$. The Rank-Score Characteristic (RSC) function, as defined by Hsu et al [1, 2], is the composite function of $s_p$ and the inverse of $r_P$. Rank-score functions map ranks to scores, and are independent of the data items. Here, the rank-score characteristic (RSC) function for the scoring system P, $f_P : N \rightarrow R$, is computed as

2 $$f_P(i)=(s_P \circ r_P^{-1})(i) = s_P(r_P^{-1}(i))$$

Similarly, $f_Q$ is computed for scoring system Q.

#### Combining two visual perception systems

Within the CFA framework [1, 2], system combination is performed either by score or rank combination. A score combination is computed as the average of the score functions, $s_p$ and $s_Q$ for each interval, $d_i$, giving us the score function of the score combination $s_{SC}$. The rank function of the score combination, $r_{SC}$, is achieved by sorting $s_{SC}$ in descending order and obtaining ranks for each $d_i$. In addition, we compute the rank combination by averaging the rank functions $r_P$ and $r_Q$, to give us the score function of the rank combination, $s_{RC}$. We sort this function in ascending order and assign ranks to get its associated rank function, $r_{RC}$ (see the example in Table 2). The performance of these combined results is measured by the distance of the newly computed points to the actual x,y coordinates where the object landed in the field.

#### Cognitive diversity between two scoring systems

**Fig. 2:**
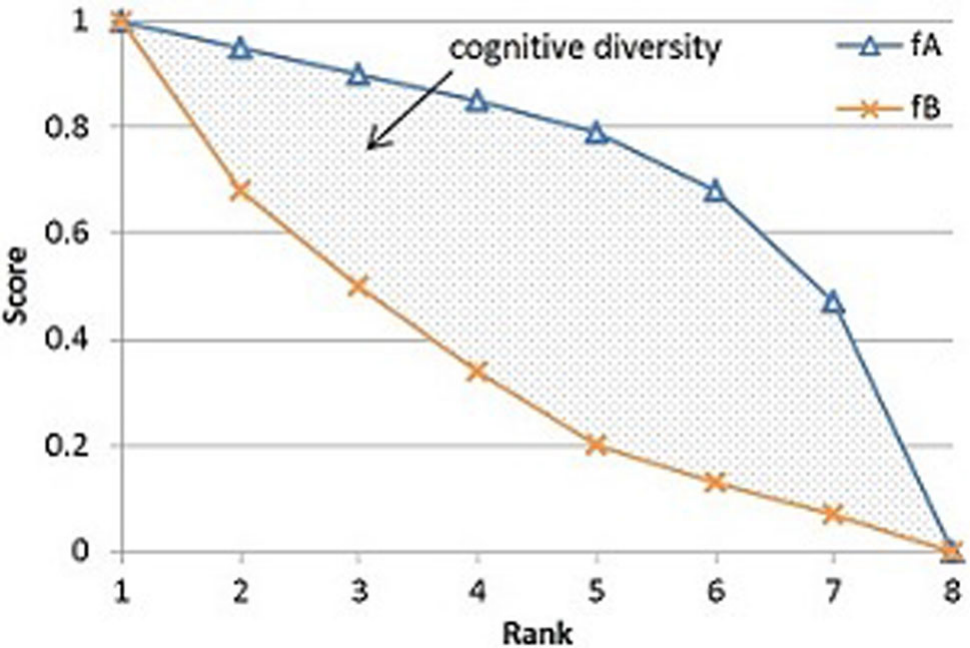
Rank-score characteristic function graph of two scoring systems,* A* and* B*

In cases where multiple scoring systems, algorithms, or approaches exist, it is beneficial to know under what circumstances combining pairs of these systems could result in improved performance. Diversity between two scoring systems A and B can be measured in a few different ways, such as the distance between score or rank functions using covariance (between $s_A$ and $s_B$) or Kendalls tau (using $r_A$ and $r_B$), respectively. Another method to measure the diversity between two scoring systems, which is used here and called cognitive diversity, is to measure the *distance* between the rank-score functions ($f_A$ and $f_B$) of the two systems [1, 2] (see formula (2) and Fig. 1). Figure 2 illustrates two RSC functions, $f_A$ and $f_B$, for two arbitrary scoring systems A and B. One distance measurement is the area between the two RSC functions. We note that the cognitive diversity between scoring systems A and B, as seen in Fig. 2, provides a powerful visualization tool on the *similarity* or *dissimilarity* between these two visual perception systems, A and B, in the context of the current study.

In this analysis, the concept of cognitive diversity is applied to the trials and scoring systems P and Q, which represent the 2 participants in a given trial pair. Therefore, the cognitive diversity of the two observers P and Q, d(P,Q), defined as the distance between the rank-score functions of two systems P and Q, $f_P$ and $f_Q$, is computed as follows:

3 $$d(P,Q)=d(f_P,f_Q) = \sqrt{ \sum _{i=1}^{m} \frac{(f_P(i) - f_Q(i))^2}{m} }$$

### Diversity rank-score function across a set of trials

Let $T = \{(p_1, q_1), (p_2, q_2), \ldots , (p_n, q_n)\}$ represent a set of n trials, each consisting of an ordered pair of participants and let $R = \{d(p_1, q_1), d(p_2, q_2), \ldots , d(p_n, q_n)\}$ represent the diversity scores for each pair in T, where $N = {1, 2, \ldots , n}$. The cognitive diversity between each pair of scoring systems, P and Q, is measured by the diversity function d(P,Q), as shown in equation (3), where m is the number of items (intervals) to be scored; in this case m is 63, indicating the number of intervals in the common visual space. The set of diversity values itself can be treated as a scoring system, making the diversity function into a diversity score function. For this purpose, the number d(P,Q), which is the diversity between scoring systems P and Q, is considered as the diversity score function value of the trial (p,q) and is denoted as $s_{(p,q)}$. The diversity rank function is attained by sorting the score function and generating ranks, giving $r_{(p,q)}$. A diversity rank-score function, $f_{(p,q)}$, is computed as

4 $$f_{(p,q)}(i)=(s_{(p,q)} \circ r_{(p,q)}^{-1})(i) = s_{(p,q)}(r_{(p,q)}^{-1}(i))$$

**Fig. 3:**
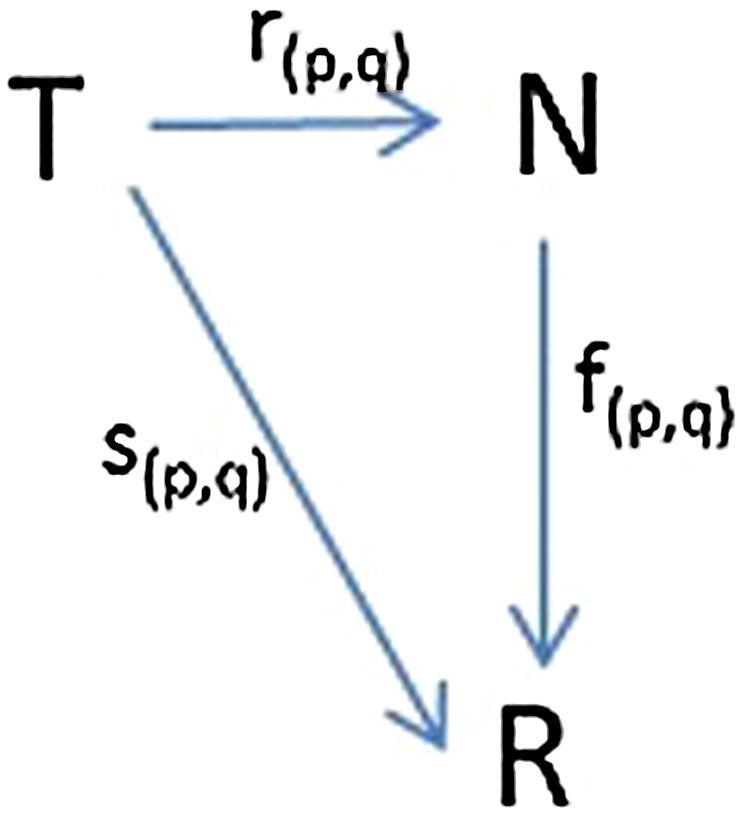
Diversity scoring system (p,q) with: (a) diversity score function $s_{(p,q)}$, (b) diversity rank function $r_{(p,q)}$, and (c) diversity RSC function $f_{(p,q)}$

The diversity rank-score function is a mapping from diversity ranks to diversity scores. The relationship between $s_{(p,q)}$, $r_{(p,q)}$, and $f_{(p,q)}$ is shown in Fig. 3.

## Case analysis using diversity rank-score graph

### Visual perception dataset

The setting for the data collection was in a grassy field in NYC’s scenic Central Park. A lab member was tasked with recruiting pairs of participants for the experiment. The pairs of subjects varied in terms of gender and relationship between the individuals. The subject pairs were randomly chosen and could be friends, siblings, husband and wife, colleagues, or acquaintances. A small metal object that was made of metal plates, nuts, and a bolt, and of size 1.5 by 1.5 inches was used for the experiment, since it was possible to throw it far distances, small enough to be hidden in the grass, and would not roll from its position once landed. The subject pairs stood 40 feet from a marked square of size 250 by 250 inches, and the individuals stood a distance of 10 feet away from each other. A member of our group tossed the metal object into the designated square. Each participant is asked individually to walk and point to where he/she believed the object landed. A marker is placed at these locations. The participants are also asked to give a measure of their confidence of his or her guess in the form of a confidence radius around the specified mark. Lab members helped the participants gauge their confidence radius by using tool consisting of 2 poles of length 36 by 36 inches to represent the x and y coordinates. Smaller radius values indicate higher confidence of the subject. A lab member measures the distance from the actual position where the object landed and the guess positions of the subjects.

The subjects are given feedback as to how far off their guess is from the actual landing point of the object. The values collected are: x,y coordinates for subject P and Q from each experiment, a confidence radius for each participant, along with the actual landing x,y coordinate of the object. All measurements are in inches. The values for the trials in this most recent experiment are shown in Table 3. Our group has conducted previous data collection activities of this type, the data of which can be found in [18].

**Fig. 4:**
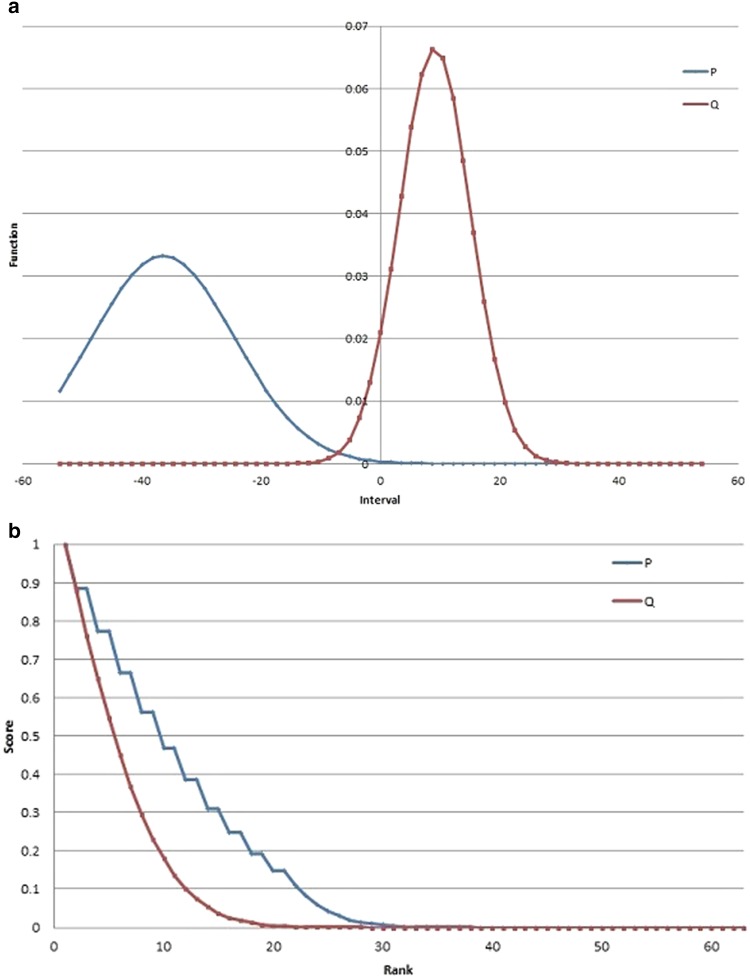
Distribution functions and Rank-score characteristic functions for P and Q in Trial 1 based on* M*_2_.** a** Distribution functions for Trial 1, based on* M*_2_.** b** Rank-score functions for Trial 1, based on * M*_2_

The distribution functions for P and Q for a sample trial are shown in Fig. 4a. Sample rank-score functions for a trial are shown in Fig. 4b.

**Table 3:** Data collected for the observed points and confidence radii for trials, along with the actual x,y coordinates

Trial	P		Confidence radius	Q		Confidence radius	Actual	
	x	y		x	y		x	y
1	126	243	12	114	287	6	120	270.5
2	69	362	8	89	358	6	85	362
3	105	220	18	60	287	10	93	321
4	93	336	10	91	285	16	81	318
5	152	170	14	141	162	16	126.5	180
6	66	250.5	16	81	288	12	88	119
7	24	314	16	31	310	8	6	313
8	94	278	12	98	220	6	86	236
9	24	235	12	23	256	12	25	240
10	96	95	8	131	71	10	107	337
11	52	187	20	97	243	16	102	269
12	107	246	10	113	233	8	113	242
13	121	191.5	10	141.5	191	8	127.5	185
14	46	277	10	73	229	8	52	254
15	73	264	18	79	267	12	84	282
16	24	442	10	23	413	10	23	432

**Table 4:** Trials ranked with respect to* M*_0_, * M*_1_, and * M*_2_

Rank	Trials (* M*_0_)	Trials (* M*_1_)	Trials (* M*_2_)
1	*d*_1_	*d*_1_	*d*_1_
2	*d*_8_	*d*_8_	*d*_3_
3	*d*_3_	*d*_2_	*d*_4_
4	*d*_4_	*d*_3_	*d*_2_
5	*d*_6_	*d*_4_	*d*_6_
6	*d*_2_	*d*_6_	**d**_**5**_
7	*d*_13_	*d*_10_	*d*_8_
8	*d*_11_	*d*_11_	*d*_7_
9	**d**_10_	*d*_14_	*d*_10_
10	*d*_4_	*d*_7_	*d*_14_
11	*d*_12_	*d*_13_	*d*_11_
12	*d*_7_	*d*_15_	*d*_15_
13	**d**_**5**_	*d*_12_	*d*_13_
14	*d*_15_	**d**_**5**_	*d*_12_
15	*d*_9_	**d**_**16**_	**d**_16_
16	**d**_**16**_	*d*_9_	*d*_9_

### Analysis results of combinations

**Fig. 5:**
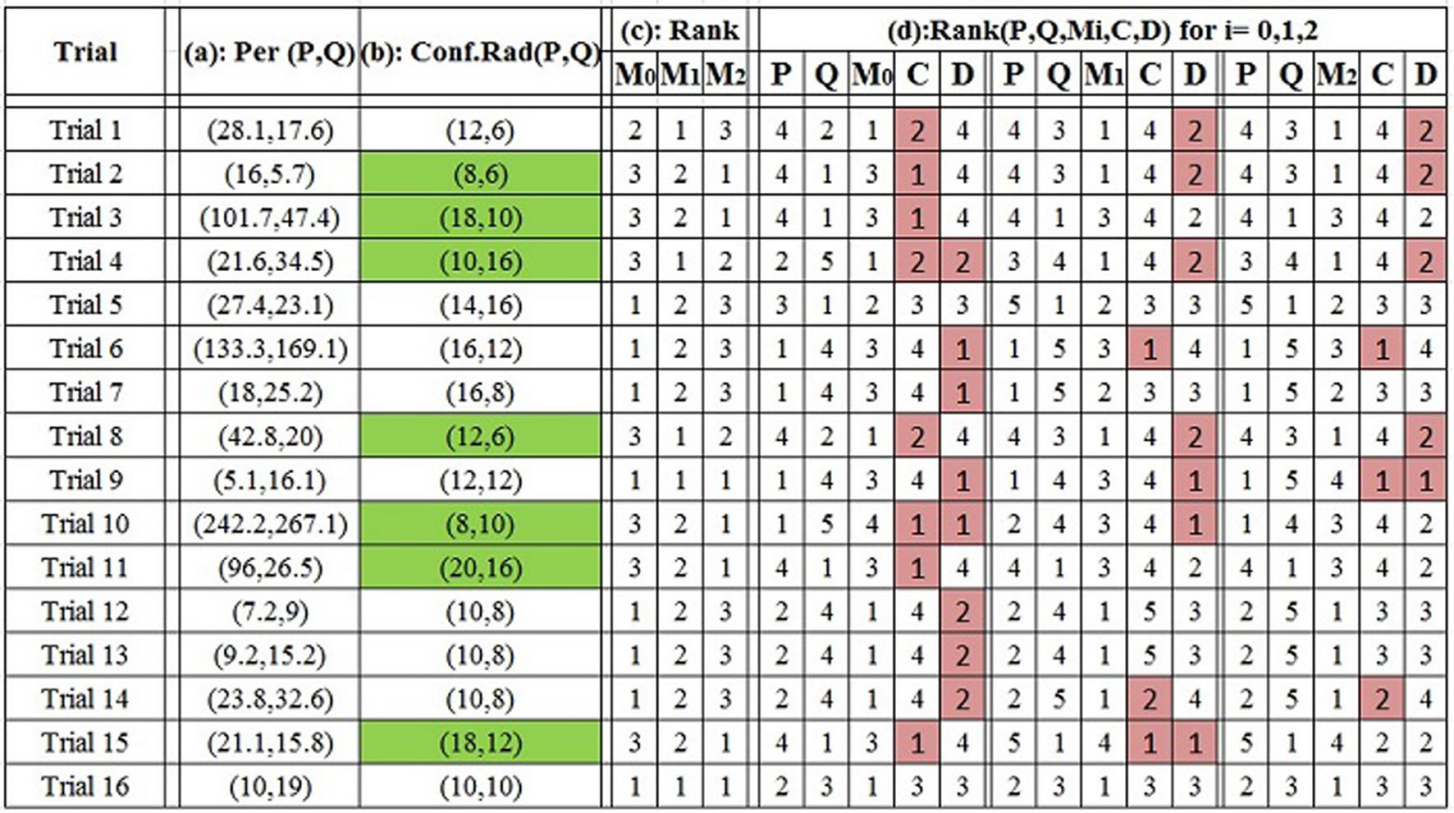
Analysis results for 16 trials [31]

The experimental results are presented in Fig. 5. The performances of P and Q, shown in column (a), are the distances to the actual landing point of the object. The confidence radii are included in column (b), in which a shaded cell indicates that the more confident participant leads to the best performance. The performance of the weighted means * M*_0_, * M*_1_, and * M*_2_ is listed in column (c). C represents the score combination and D represents the rank combination. The last column, (d), presents information for the results using each of the weighted means, along with the score and rank combinations (C and D). For each $i=\{0,1,$ and $2\}$, P, Q, * M*_*i*_, C, and D are ranked in descending order of performance; repeated ranks indicate tied performance. Rank 1 showed the best performance, meaning the closest interval to the actual location of the object. Cases where the score (C) or rank (D) combinations either outperformed or tied the best individual system are highlighted.

**Fig. 6:**
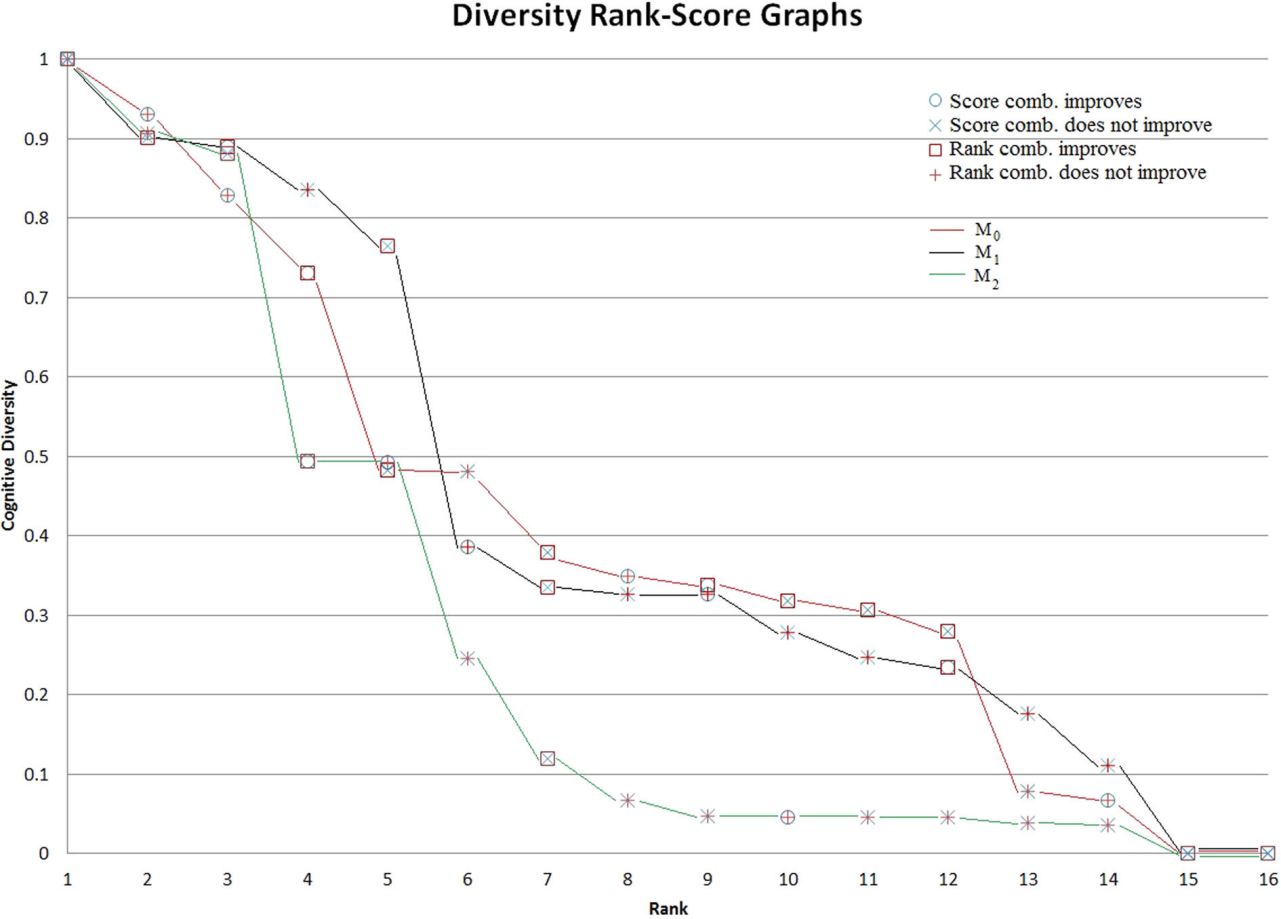
Diversity rank-score graphs based on * M*_0_, * M*_1_, and * M*_2_, respectively

### The role of diversity rank-score graphs

After performing the score and rank combinations for the three different computations of M (* M*_0_, * M*_1_, and * M*_2_), we can summarize the results as follows: Using * M*_0_, the score and/or rank combination for 14/16 trials showed either tied or improved performance compared to the best individual system; using * M*_1_, 9/16 trials; and using * M*_2_, 7/16 trials. The diversity rank-score functions for the scoring systems created according to the three different computations of the mean: * M*_0_, * M*_1_, and * M*_2_, are depicted in Fig. 6. Examination of these graphs, along with the performance of the corresponding system pair combinations, can help us understand the role of cognitive diversity in system combinations by score and rank. To make the connection with the trials, Table 4 is included to show the ranking of trials according to the diversity of their component scoring systems, for each case of * M*_0_, * M*_1_, and * M*_2_. When comparing with the performances of the system combinations, we detect a tendency for pairs of systems with relatively high diversity to have more improved performance. In this study, this observation is most strongly supported by the data in the * M*_1_ scenario. In new situations, where we may not be able to predict the performance, analyzing the relative diversities between scoring systems may give us insight into which pairs of systems are most likely to show improvement with combination.

We observe that the diversity rank-score graphs are good indicators for the combination outcome. For example, trials * d*_5_ and * d*_16_ appear at the very end of the graph in * M*_0_, * M*_1_, and * M*_2_ (see Figure 6 and Table 4). In these two trials, neither rank nor score combination helps improve the outcome. However, even though trial * d*_9_ has a very low diversity (Table 4), its combination of scoring systems P and Q is better than or equal to the best of P and Q since P has a relatively high performance.

## Conclusion and further work

In this paper, we studied the combination of multiple visual perception systems using the CFA framework and the diversity rank-score function. By establishing each visual perception system as a scoring system on a set of options (possible locations, in our context) in a common visual space, the problem of combining multiple visual perception systems is treated as a problem of combining multiple scoring systems. Using a dataset of an experiment with sixteen trials where each trial consists of a pair of two observers, we studied various issues as to how the diversity between these two observers (and their individual perception systems) affects the performance of the combined system.

At the individual trial level, we illustrated that the rank-score characteristic (RSC) function graphs of the two scoring systems (perception systems) can provide a useful visualization tool on the *similarity* or *dissimilarity* between these two visual perception systems (see Fig. 2 and Sect. 2.2.3). At the population level, the diversity rank-score graphs on three common visual space definitions, * M*_0_, * M*_1_, and * M*_2_, respectively provide a powerful visualization comparison, not only among all (sixteen) trials in an experiment, but also among all (three) analytic methods based on * M*_0_, * M*_1_, and * M*_2_, respectively (see Fig. 5 and Sect. 2.3). Our current study suggests a few issues which are worthy of further investigation. We list three here:

1. With the diversity rank-score function defined in formula (4) and the diversity rank-score graphs based on * M*_0_, * M*_1_, and * M*_2_, extend the study to include higher order of * M*_*i*_, i = 4, 5, and so on (refer to formula 1).
2. Establish a CFA framework to study the combination of more than two visual perception systems. In this regard, the notion of *diversity* among more than two systems would have to be defined differently.
3. Apply the visualization tool illustrated in current work to combination of multiple sensing systems, multiple robotics systems, and multi-modal physiological imaging systems such as MRI, EEG, and EKG.
